# Maximum-likelihood determination of anomalous substructures

**DOI:** 10.1107/S2059798317013468

**Published:** 2018-02-01

**Authors:** Randy J. Read, Airlie J. McCoy

**Affiliations:** a Department of Haematology, University of Cambridge, Hills Road, Cambridge CB2 0XY, England

**Keywords:** likelihood, single-wavelength anomalous diffraction, substructure determination

## Abstract

A fast Fourier transform (FFT) method is described for determining the substructure of anomalously scattering atoms in macromolecular crystals that allows successful structure determination by X-ray single-wavelength anomalous diffraction (SAD). This method is based on the maximum-likelihood SAD phasing function, which accounts for measurement errors and for correlations between the observed and calculated Bijvoet mates. Proof of principle is shown that this method can improve determination of the anomalously scattering substructure in challenging cases where the anomalous scattering from the substructure is weak but the substructure also constitutes a significant fraction of the real scattering. The method is deterministic and can be fast compared with existing multi-trial dual-space methods for SAD substructure determination.

## Abstract 2

Likelihood-based SAD substructure determination can be initiated using a fast translation-search algorithm based on a linear approximation to the SAD likelihood target, followed by log-likelihood-gradient map completion.

## 1. Introduction   

Single-wavelength anomalous diffraction (SAD) phasing has become the predominant method to solve novel structures when a molecular-replacement approach is not possible or not sufficient (Hendrickson, 2014 ▸). Contributing to the rise of SAD as the experimental phasing method of choice have been enhanced methods for optimizing the selection and scaling of data from multiple crystals (Liu *et al.*, 2012 ▸; Foadi *et al.*, 2013 ▸; Akey *et al.*, 2016 ▸; Terwilliger *et al.*, 2016*a* ▸,*b* ▸), and effective methods for correcting for radiation damage (Borek *et al.*, 2013 ▸).

Given data with enough anomalous signal, SAD phasing is bootstrapped from a hypothesis concerning the position of as little as a single atom in the structure, to which atoms are progressively added. The full substructure is usually considered to be all of the atoms with significant anomalous scattering, but the substructure can also include atoms that have insignificant anomalous scattering, such as a partial protein or nucleic acid model located by molecular replacement. When the substructure is sufficiently complete, the phases derived from the substructure become good enough that density modification, model building and refinement can be used to add atoms to the structure without reference to the anomalous differences, at which point the structure is, by convention, no longer called a substructure (McCoy & Read, 2010 ▸).

Locating the initial one or more atoms in the anomalous substructure is the linchpin of the SAD phasing bootstrap. Currently, hypotheses for initializing the substructure are generated using methods adapted from small-molecule crystallography, typically treating the anomalous differences as if they were the raw diffraction observations. The *SOLVE* program (Terwilliger & Berendzen, 1999 ▸) ranks grid locations of the first one or two atoms against the vector minimum function of the anomalous difference Patterson (Buerger, 1970 ▸; Terwilliger *et al.*, 1987 ▸); further sites are added from analysis of anomalous difference Fourier maps, with various metrics and automated decision making used to identify and pursue good substructures.

In the multi-trial direct-methods approach pioneered by *MULTAN* (Germain *et al.*, 1970 ▸) and *RANTAN* (Yao, 1983 ▸), subsets of reflections are assigned phases and these are used to initiate direct-methods phasing of the anomalous differences. However, reciprocal-space direct methods alone tend to lose enantiomorph discrimination, causing the problem of the false ‘U-atom’ solution, especially for larger structures. This can be ameliorated by enforcing atomicity in real space, in dual-space algorithms. The first of these dual-space algorithms to be developed, *Shake-and-Bake* (Miller *et al.*, 1993 ▸), derives initial phases from randomly generated initial atomic coordinates, and these are then refined in cycles alternating between reciprocal-space direct methods (optimizing the minimal function) and real-space peak picking from Fourier maps with a minimum peak (atom) separation distance. *SHELXD*, which was developed subsequently (Schneider & Sheldrick, 2002 ▸), employs a similar dual-space approach but seeds the process with better-than-random phases. Peaks from a sharpened anomalous difference Patterson are taken as two-atom separation vectors, and the oriented atom pairs are placed in the unit cell with vector-scoring functions (Nordman, 1966 ▸). The substructure is then expanded to the expected number of sites with more anomalous difference Patterson analysis, dual-space recycling (using the tangent formula to refine phases in reciprocal space) and random omit procedures. *HySS* (Grosse-Kunstleve & Adams, 2003 ▸) modifies the *SHELXD* algorithm so that the initial oriented two-atom substructures from Patterson analysis are placed in the unit cell with the fast translation function, achieving expansion to three sites by fixing the two-atom substructure and using a second fast translation function to search for a single atom; phase refinement using the tangent formula in reciprocal space is replaced by the related procedure of density squaring in real space. The multi-trial nature of the dual-space algorithms is computationally intensive for challenging cases, and it is not uncommon to obtain only a single solution in thousands of trials (Sheldrick, 2010 ▸). If the data have weak anomalous signal and/or there are many anomalously scattering sites, substructure determination remains a bottleneck in SAD phasing, even when there is sufficient signal that phasing would succeed with a correct substructure.

An interesting alternative to conventional direct methods and the associated dual-space algorithms is to apply charge-flipping algorithms to the anomalous differences. Dumas & van der Lee (2008 ▸) demonstrated that *Superflip* (Palatinus & Chapuis, 2007 ▸) could be effective even in solving large substructures.

If a sufficiently complete anomalous substructure can be obtained, which would normally mean that the substructure accounts for the majority of the anomalous scattering, it can be used to phase the structure with the maximum-likelihood SAD (MLSAD) function (McCoy *et al.*, 2004 ▸; Pannu & Read, 2004 ▸). MLSAD is based on the joint probability distribution of a Bijvoet pair of diffraction observations, conditional on the corresponding pair of structure-factor contributions calculated from a substructure model. Because MLSAD includes the contribution from the real scattering, the phase ambiguity that arises from considering only the anomalous component of the scattering is partly broken. A significant component of the success of MLSAD has been the use of log-likelihood-gradient maps (McCoy & Read, 2010 ▸; Read & McCoy, 2011 ▸), rather than anomalous difference Fouriers, to edit and complete the substructures, an approach introduced in *SHARP* (de La Fortelle & Bricogne, 1997 ▸). Recently, it was shown that substructure determination could be significantly strengthened by giving a prominent role to MLSAD throughout the process, rather than just in adding weak sites to the bulk of the substructure (Bunkóczi *et al.*, 2015 ▸); *HySS* was modified so that MLSAD log-likelihood-gradient substructure completion took over after as few as two sites had been determined.

Despite the improvements in substructure building and SAD phasing with maximum-likelihood methods, SAD phasing is still reliant on random or Patterson-based methods to seed the placement of the first atoms in the phasing bootstrap. Missing from the repertoire of methods for initializing the atomic substructure is a maximum-likelihood approach. Since determining the substructure remains a bottleneck in structure determination by SAD, and since maximum-likelihood methods have an established record in improving methods in other aspects of macromolecular crystallography, we expected that maximum-likelihood approaches should be able to improve the speed and reliability of substructure determination.

We describe here an approximation of the MLSAD target, termed *Phassade* (for ***Ph**aser* **a**nomalous **s**ub**s**tructure **de**termination), that can be calculated by fast Fourier transform (FFT) to generate a set of trial positions starting from a null substructure. Effectively, this method simultaneously tests hypotheses for all potential positions for an anomalous scatterer on a grid covering the unit cell. The trial positions can be refined with the exact MLSAD target and then used to seed structure completion by log-likelihood-gradient maps. The *Phassade* search target retains the strength of the MLSAD target in automatically combining information from both the real and imaginary scattering contributions, and hence improves on current methods when the anomalous signal is low but the real contribution to the scattering is high, for example when the anomalous scatterer is a metal ion and the wavelength is far from the absorption edge.

## 2. Initiating likelihood-based substructure determination   

The existing MLSAD log-likelihood-gradient completion functions require a starting point. For an empty substructure, the (complex) derivatives of MLSAD are all zero because the effect of changes in the calculated structure factors will be identical for shifts in opposite directions. When the substructure is not empty, the effect of changes in the calculated structure factors will be different for shifts in different directions in the complex plane because these will have different effects on the amplitudes.

The success of molecular replacement using fragments as small as single atoms (McCoy *et al.*, 2017 ▸) inspired a new way to think of the problem of locating the first atom by SAD. Single-atom molecular replacement uses the likelihood-based fast translation function (McCoy *et al.*, 2005 ▸) to score possible positions for the atoms with a single FFT. This fast translation search is based on a linear approximation of the molecular-replacement likelihood target, expressed in terms of the calculated intensity as a function of translation. We reasoned that if the SAD likelihood function were expressed in terms of calculated intensities, the same approach could be applied to search for positions for anomalous scatterers even when there is no starting structure.

### 2.1. Unphased SAD likelihood target   

**Figure 1:**
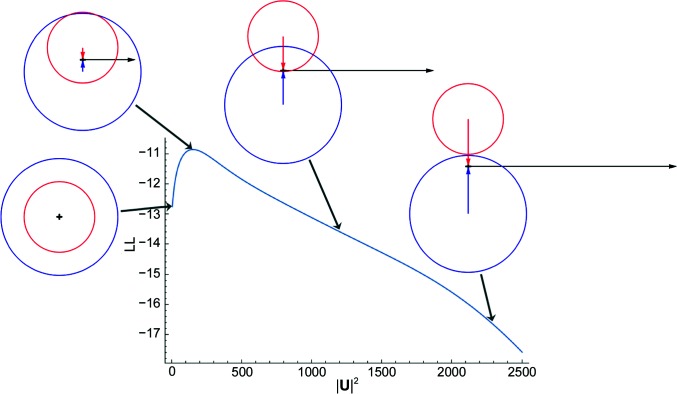
SAD likelihood function for the (8, 15, 21) reflection in the tryparedoxin test case, as a function of |**U**|^2^. Grey arrows pair diagrams illustrating the Harker constructions for particular values of |**U**|^2^ with the corresponding points on the curve. In each Harker construction, the black arrow indicates the real component of **H** ^+^ and **H** ^−*^, whereas the blue and red arrows indicate their respective imaginary components. The blue and red circles, with radii corresponding to *F* ^+^ and *F* ^−^, respectively, represent the possible complex values of **F** ^+^ and **F** ^−^.

The exact version of the required target can be computed by an adaptation of the methods used to compute log-likelihood-gradient MLSAD maps. The MLSAD target is a function of the structure-factor amplitudes for the observed Bijvoet pair, as well as the corresponding calculated structure factors, **H** ^+^ and **H** ^−*^ (the complex conjugate of the structure factor for the minus hand) and variance terms. If the substructure is composed of a single type of anomalous scatterer with scattering factor *f* + *if*′′ (where *f* = *f*_0_ + *f*′), **H** ^+^ and **H** ^−*^ can be expressed in terms of a single structure factor, **U**, computed from point atoms of unit weight. If we assume that all atoms have unit occupancy and a *B* factor estimated from the Wilson distribution, a simple equation for **U** applies,
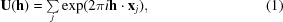
where the sum is over all atoms in the unit cell. This can be modified to account for varying occupancies and for *B* factors differing from the mean,
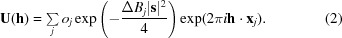



The pair of calculated structure factors is then obtained by taking account of the scattering factors for the two Friedel mates and the overall Wilson *B* factor, 
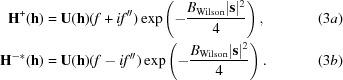



Note that a shift in the phase of **U** causes identical shifts in the phases of **H** ^+^ and **H** ^−*^ but, since the evaluation of the MLSAD likelihood function involves integrating over all possible phases for the corresponding true structure factors, the value of the likelihood target is unchanged. (Visualized in terms of the Harker construction for phasing, the geometrical relationship between **H** ^+^ and **H** ^−*^ is unchanged, as is the degree of overlap of the circles, but the whole construction is rotated.) For this reason, the MLSAD likelihood function can be defined in terms of *U* ^2^ = |**U**|^2^. The phase assigned to **U** is therefore arbitrary, so for convenience it can be taken as purely real. Fig. 1 ▸ illustrates the variation of the log-likelihood MLSAD target as a function of *U* ^2^, along with the Harker constructions for purely real **U** that correspond to several points along the curve.

### 2.2. Computing a fast approximation to the unphased SAD likelihood target   

The molecular-replacement likelihood-enhanced fast translation function (McCoy *et al.*, 2005 ▸) is based on a linear approximation of the molecular-replacement likelihood target as a function of the calculated intensity, derived as a first-order Taylor series approximation centred on the expected value of the calculated intensity. Similarly, the *Phassade* fast SAD translation function can be derived from a Taylor series approximation to the MLSAD likelihood target centred on the expected value of *U* ^2^. If the logarithm of the MLSAD likelihood target is denoted *L*, then 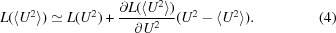



As noted above, **U** can be treated as a purely real quantity *U*, which simplifies the expression for the derivative required for the linear approximation,
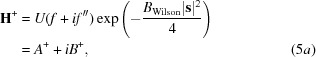
where
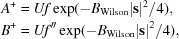
and
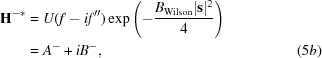
where
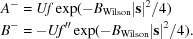



**Figure 2:**
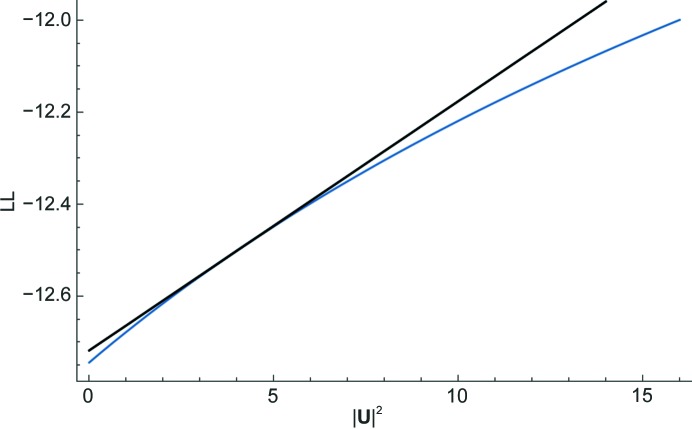
Expanded view of the likelihood function shown in Fig. 1 ▸, emphasizing the region likely to be encountered in a search for one fully occupied Se atom. The linear approximation in black is centred on the expected value of |**U**|^2^, which is equal to the number of symmetry operators in space group *P*2_1_2_1_2_1_, *i.e.* four.

The derivative for the slope of the linear approximation is found using the chain rule, expressed in terms of partial derivatives that are already required for refinement of the substructure against the MLSAD likelihood target (McCoy *et al.*, 2004 ▸) or for computing log-likelihood-gradient maps (McCoy & Read, 2010 ▸),
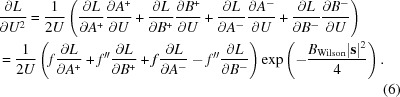



Equation (6) here is closely related to equation (6) from McCoy & Read (2010 ▸) when expressed in terms of a purely real *U*. Centring the linear approximation on the expected value of *U* ^2^ ensures that it is most accurate for the values that will be encountered in a translation search. The expected value can include the contribution of an existing partial structure, which will be set to zero when searching for the first atom in the substructure. The contribution to the expected value of the atom being placed by the translation search, plus its symmetry copies, is simply equal to the number of symmetry operators times the expected intensity factor (a statistical factor ∊ that is usually 1) for the reflection, weighted by the occupancy assumed for the atom being placed, 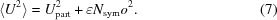



Fig. 2 ▸ illustrates the linear approximation for the same case as shown in Fig. 1 ▸, focusing on the values of *U* ^2^ likely to be encountered for a substructure with a single fully occupied unique atom and centred on the corresponding expected value of *U* ^2^, *i.e.* the number of symmetry operators.

The linear approximation to the MLSAD target can be computed, for all potential positions of a unique anomalous scatterer, with a single FFT by using the algorithm of Navaza & Vernoslova (1995 ▸). In this framework, the partial structure factor for each symmetry-related copy of the search atom is simply a real number corresponding to the search occupancy for the atom. Once a trial position for an atom has been selected from a peak in the FFT, the MLSAD target can be used to refine the resulting anomalous-scatterer model, including occupancies, *B* factors and variance terms.

### 2.3. Variance terms used in the search   

Computing the MLSAD target requires estimates for the variance terms relating to the fractions of the real and imaginary scattering accounted for by the model. Unlike the molecular-replacement case, in which one usually has reasonable confidence in a prior estimate of the total ordered scattering of the asymmetric unit of the crystal (and there are only discrete options corresponding to integer numbers of molecules), there is considerable uncertainty in the prior knowledge about the amount of scattering from anomalous scattering. This is particularly an issue for soaking experiments, but even for selenomethionine phasing there is a good chance that one or more methionine residues will be poorly ordered. The variance parameters can be refined, for a null model or after placing additional atoms in the substructure, but it is difficult to predict precisely which fraction of the variance will be accounted for when an atom in the substructure is placed correctly.

In molecular-replacement searches in *Phaser* (Storoni *et al.*, 2004 ▸; McCoy *et al.*, 2005 ▸), the variance terms are reduced by the fraction of scattering that is expected to be explained. However, in this work we have not yet attempted to adjust the refined variances for the effect of placing an additional atom in the substructure.

### 2.4. Occupancy of the search atom   

In substructure determination, there can be considerable *a priori* uncertainty about the occupancy of the anomalous scatterer being placed, especially for soaking experiments or bound halides. Visualized in terms of the Harker construction, varying the occupancy of an atom being placed, scales the relative shift of the circles corresponding to the two diffraction observations; a small shift in the direction that will maximize the likelihood target for an optimal choice of occupancy will nonetheless increase the likelihood score, whereas a shift that is too large may even result in a reduction of the likelihood score. Such considerations suggest that it may be preferable to carry out the search using smaller occupancies than those expected for atoms in the substructure. In the limit of an infinitesimal occupancy, such a search corresponds to a log-likelihood gradient calculation. One advantage of using low initial occupancies for the search atoms is that it becomes unnecessary to worry about the reduction in the variance terms that should occur when an atom is correctly placed. The search occupancy is an adjustable parameter in the current version of the *Phassade* algorithm.

## 3. Completing a partial substructure   

Because the *Phassade* target is based on a linear approximation that can include the contribution of a fixed background substructure, it is possible to complete a partial substructure by using *Phassade* to select one or more new atoms at a time. Alternatively, the log-likelihood-gradient completion algorithm (Read & McCoy, 2011 ▸) can be employed, starting from a substructure containing as little as a single unique atom. The two approaches should yield similar results, but they differ in the sense that the *Phassade* target evaluates the effect of including an atom with a defined occupancy at a particular site, whereas the log-likelihood-gradient map evaluates the effect on the likelihood target as an anomalous-scatterer occupancy is increased infinitesimally at each site. As the assumed occupancy for *Phassade* approaches zero, the two approaches should converge. These considerations provided a second reason to explore different choices of assumed occupancy in the test calculations.

For non-enantiomorphic space groups, the search for the first atom will yield pairs of positions related by inversion, corresponding to the two possible hands for the substructure and specifying a choice of origin. Depending on the symmetry and whether or not this atom is on a special position, the substructure may be centrosymmetric, in which case the search for the next atom will also yield pairs of positions related by inversion. To break the centrosymmetry and avoid mixing solutions corresponding to different choices of hand, it is necessary to add new atoms to the substructure one by one until the centrosymmetry is broken. At this point, multiple atoms can be added simultaneously if there is more than one significant peak in the search.

## 4. Test calculations   

Several test cases were used during development to establish sensible defaults and to gauge the performance of the new algorithm, which was benchmarked against the current *HySS* algorithm that includes *Phaser* log-likelihood-gradient completion (Bunkóczi *et al.*, 2015 ▸). The cases were chosen to sample substructures with different levels of anomalous signal and to evaluate the effect of accounting for the real scattering contribution of the anomalous scatterers. These tests provide a proof of principle that the method works, but an exhaustive characterization against a large test set that was not used in training has yet to be carried out.

### 4.1. Selenomethionine in tryparedoxin   

The structure of tryparedoxin-I from *Crithidia fasciculata* (PDB entry 1qk8; Alphey *et al.*, 1999 ▸) was originally determined by multi-wavelength anomalous diffraction using a selenomethionine derivative, but it is possible to solve it using just the data from the peak wavelength (0.9790 Å; Bunkóczi *et al.*, 2015 ▸). There is only one Se site, corresponding to the single ordered methionine in the structure. The *Phassade* search for a fully occupied Se atom yields a single unique peak with a *Z*-score of 20.6; this site refines to a final log-likelihood gain (LLG) of 575. The entire calculation, including the final phasing, takes a total of 1.9 s on a Mac Pro with a 3.5 GHz Xeon processor. The substructure can also readily be determined with a default run of *HySS*, taking a total of 13.6 s without the final phase calculation.

Achieving a clear signal does not require placing a Se atom at full occupancy. In fact, searches using occupancies ranging from 0.05 to 1.0 all give very similar results in terms of the signal to noise and run time.

### 4.2. Hen egg-white lysozyme iodide soak   

This test used data collected on a copper rotating-anode source from a tetragonal form crystal of hen egg-white lysozyme that had been soaked in 0.5 *M* potassium iodide; these data are distributed for *CCP*4 and *PHENIX* tutorials on experimental phasing in *Phaser* (http://www.phaser.cimr.cam.ac.uk/index.php/Tutorials). The refined iodide occupancies for the 14 atoms in the correct substructure solution range from 0.11 to 0.73. When the search occupancy is set too high, the signal to noise of the search is reduced substantially, at least partly because of noise piling up on special positions. For instance, when the search occupancy is set to 1, the largest features in the map are holes, with the deepest hole (on a twofold axis) having a *Z*-score of 28.3. The peaks in this map suggest six potential solutions for the first atom. Of these, the second in the list (*Z*-score of 6.8) corresponds to the iodide site with highest occupancy in the final substructure; it refines to a final LLG of 127.5. The first peak (*Z*-score of 7.0) is also correct, although it is a weaker site that refines to an LLG of 76.3, but the remaining four are incorrect. In contrast, search occupancies of 0.6 or less yield a single dominant site, which corresponds to the atom with highest occupancy in the complete substructure. As the search occupancy is reduced, the deepest holes in the fast SAD translation search map become shallower and the signal to noise improves, with lower occupancies yielding a *Z*-score of 8.8.

Space group *P*4_3_2_1_2 is enantiomorphic, so there is no hand ambiguity in the substructure search. Once the first site has been placed, the origin is defined and it is possible to add multiple new sites found as significant peaks in new searches. Substructure completion can be carried out with either the *Phassade* search or log-likelihood-gradient completion, both of which find the additional sites with very clear discrimination from noise. The log-likelihood-gradient completion algorithm has been highly optimized, so it yields a complete solution more quickly in the current implementation.

Finding the first site with the *Phassade* search takes 2.3 s, using a search occupancy of 0.05, and placing the remaining 13 sites with log-likelihood-gradient completion takes an additional 9.1 s, for an overall total of 11.4 s. By comparison, a default run of *HySS* takes 58.7 s to determine a substructure with 14 sites, four of which are discarded during phasing and log-likelihood-gradient completion calculations in *Phaser*, to obtain the same substructure found with the new approach.

### 4.3. *Clostridium acidurici* ferredoxin   

The structure of *Clostridium acidurici* ferredoxin was refined against data collected to 0.94 Å resolution (PDB entry 2fdn; Dauter *et al.*, 1997 ▸), starting from a structure previously determined at 1.84 Å resolution (PDB entry 1fdn; Duée *et al.*, 1994 ▸). Data were collected with a wavelength of 0.883 Å, with no attempt being made to optimize the anomalous signal from the Fe atoms in the two Fe_4_S_4_ clusters in this protein. As a result, the anomalous signal is weak although detectable, and it is very difficult to determine the substructure using conventional methods based on the use of the anomalous differences. Note that there is very little anomalous signal beyond about 2 Å resolution, whereas each Fe atom accounts for nearly 4% of the total real scattering at around 1 Å resolution, near the limit of the data.

*HySS* only succeeds in solving the substructure when the new algorithms employing *Phaser* log-likelihood-gradient completion are employed, thus taking account of the real component of the scattering in the completion phase. A successful run takes 1105 s to find all eight Fe atoms and all 16 S atoms in the structure, as well as 19 low-occupancy sites corresponding to well ordered C, N and O atoms.

A preliminary test of single-atom molecular-replacement methods (McCoy *et al.*, 2017 ▸) showed that there is sufficient signal in just the real scattering contribution of the Fe atoms to atomic resolution to place them reliably. With the *Phassade* search, it is not necessary to choose whether to pay attention to just the real or imaginary components of scattering. Indeed, a search for the first Fe atom with the fast SAD translation search gives a dominant single solution with a *Z*-score of 17.5 and an LLG of 106.4 in 8.0 s. As for the lysozyme test case, placing a single atom in space group *P*4_3_2_1_2 defines both the hand and the origin.

The log-likelihood-gradient completion can search for additional Fe atoms or for a combination of atom types, and when a combination of atom types is used the likelihood score can be used to distinguish the correct hand. Two tests for completion were carried out. The first test searched for additional Fe or S atoms, testing both *P*4_3_2_1_2 and its enantiomorph *P*4_1_2_1_2, and was restricted to two cycles of completion. The search in *P*4_3_2_1_2 found a total of 27 sites, six of which were labelled as Fe and 21 as S, with a final LLG score of 6094. In contrast, the search in *P*4_1_2_1_2 found a total of 33 sites, 17 of which were labelled as Fe and 16 as S, but even with a larger number of sites the final LLG score was only 5299. This run, testing the space group and its enantiomorph, took 172.3 s, for an overall total of 180.3 s, compared with 1105 s for the *HySS* calculation that found a similar number of sites but did not resolve the choice of hand. The second test searched for additional Fe, S or N atoms (with N atoms serving as proxies for C, N or O) and carried on until no further changes were made in the substructure, taking 1115.9 s to search in both space groups. The search in *P*4_3_2_1_2 found a total of 388 sites, eight of which were labelled as Fe, 40 as S and 340 as N, with a final LLG score of 32 304, whereas the search in *P*4_1_2_1_2 found a total of 395 sites, 15 of which were labelled as Fe, 367 as S and 13 as N, with a final LLG score of 25 516. In the deposited PDB file there is a total of 564 records for non-H atoms, including all solvent atoms and alternate conformers. Note that the weak anomalous signal was sufficient to distinguish clearly between the choices of hand and assisted in the correct identification of the element types. Nonetheless, the real scattering signal dominates in this case to the extent that essentially the correct atomic positions can be obtained in the wrong hand.

### 4.4. Carbamoylphosphate synthase large subunit from *Exiguobacterium* species 255-15   

Carbamoylphosphate synthase (PDB entry 2pn1) is an unpublished structure determined by the Joint Center for Structural Genomics using two-wavelength selenomethionine MAD phasing. It is possible to solve this structure by SAD phasing using the data from either wavelength, but it is much more difficult with the high-energy remote data set (wavelength of 0.91837 Å) used in the tests reported here. A substructure containing all seven Se sites can be determined with *HySS* in 1171 s when the *Phaser* log-likelihood-gradient completion algorithm is used, but not when *HySS* is confined to the earlier direct-methods approaches (Bunkóczi *et al.*, 2015 ▸).

Using the current default protocol, the *Phassade* search fails to solve this substructure. A default search for the first atom in the substructure yields a single dominant solution for an atom about 1 Å from a crystallographic twofold. By reducing the thresholds to preserve a longer list of potential solutions, a list of five one-site solutions including a correct solution (No. 4 in the list) can be obtained in 10.1 s. In space group *C*2 single-atom substructures are always centrosymmetric, so it is necessary to add atoms one by one to avoid adding pairs that preserve the centre of symmetry, until this symmetry is broken. Starting from the correct single site found in the more exhaustive search for the first atom, a default search with *Phassade* finds three potential solutions in 216.5 s; the first of these, with an LLG score of 170.0, is correct, whereas the other two solutions (LLG values of 165.7 and 156.1) each have one incorrect position, failing to place the Se atom with the highest *B* factor in the refined structure. A fairer test is to start a branched search from all five potential solutions for the first atom, in which case the same three potential solutions are found in 2569 s.

## 5. Discussion   

### 5.1. Comparison with methods relying on the estimation of *F*_A_   

Current methods for substructure determination are built upon the estimation of *F*_A_, the structure factors of the anomalously scattering atoms, through Pattersons calculated from the square of the coefficients and/or direct methods using the *F*_A_ estimates directly. The vast majority of anomalous substructure determinations use the Rossmann approximation (Rossmann, 1961 ▸; Hendrickson, 2014 ▸),
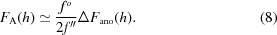



This approximation is only valid if the anomalous scattering effects are relatively small and it can be assumed that the modulus of normal scattering can be taken as the average of the square root of the intensities of the Bijvoet pairs. The approximation overestimates *F*_A_ for structure factors for which *F*_PH_ and *F*_A_ are in phase, since (8) approximates an expression that includes the sine of the phase difference,
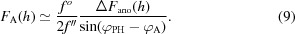



The sine term introduces noise, and peaks in the anomalous difference Patterson will be half weight (Rossmann, 1961 ▸). In addition, if only SAD data are available, this approximation does not reflect any contribution from the real scattering by anomalous scatterers.

If isomorphous differences are also known, such as from a MAD experiment, then the information that they give is complementary and they can be combined to give better estimates of *F*_A_. *F*_A_ can be estimated by solving a set of simultaneous equations (Hendrickson, 1985 ▸). Despite the estimates of *F*_A_ being more robust when MAD data are available, in practice they can be affected by radiation damage, which tends to be severe when anomalously scattering atoms are present and absorbing energy, and by other systematic errors, such as those in scaling. Terwilliger (1994 ▸) showed that a Bayesian analysis of the MAD data, applying prior probabilities to the *F*_A_ estimates based on the expected scattering, improved estimates of the *F*_A_ in the presence of significant errors.

The *Phassade* search avoids any requirement to estimate *F*_A_, as the SAD likelihood target is based directly on the joint probability distribution of the Bijvoet pair of structure factors. This target automatically takes account of the effects of both real and imaginary scattering in the atoms comprising the substructure, so it is not necessary to determine in advance which contribution to the signal will be important. As a result, it will succeed for substructures in which a substantial part of the signal comes from the real scattering contribution, such as the ferredoxin case discussed here, as well as those for which the anomalous scattering contributions are very large.

### 5.2. Comparison with direct methods   

It is perhaps surprising that a method completely ignoring correlations among triplets of reflections, which have been thought to be essential to the most powerful substructure-determination methods, can be as successful as it is. This is despite the current algorithm being completely deterministic, being built on a systematic (albeit branched) search. The implication is that what has been given up in ignoring these correlations has been, at least in large part, recovered by accounting much more rigorously for statistical effects, in particular the propagation of measurement errors and errors from model incompleteness.

### 5.3. Future directions   

As it stands, the combination of the *Phassade* search and log-likelihood-gradient completion with the SAD likelihood target is already competitive with existing methods for data sets with reasonably clear signal and relatively modest numbers of sites. However, there is certainly room to take inspiration from some of the approaches that have enhanced the power of dual-space methods. It is not necessary to be restricted to searching for single atoms; in both *SHELXD* (Schneider & Sheldrick, 2002 ▸) and *HySS* (Grosse-Kunstleve & Adams, 2003 ▸) peaks selected from the anomalous difference Patterson map are used to prime the search for pairs of atoms separated by the corresponding vectors.

For particularly difficult cases, adding a stochastic element to the search could be helpful, as has been found for the dual-space methods. For example, the random deletion of a subset of sites, followed by re-expansion, extends the power and accuracy of substructure determination in *SHELXD* (Schneider & Sheldrick, 2002 ▸).

Further automation will be achieved by combining the *Phassade* search for the first atoms (or pairs of atoms) with log-likelihood-gradient completion in a single task. For robustness, it would be essential to avoid adding multiple sites at once as long as the substructure is centrosymmetric, but efficiency would be gained by allowing multiple sites to be added simultaneously after the centrosymmetry has been broken.

We expect that these and other developments of the maximum-likelihood approach to substructure determination will further enhance the robustness, power and convenience of the method. When the algorithms have been validated by tests on a wider range of data, they will be incorporated into official releases of the *Phaser* software.
